# Aligned TiO_2_ Scaffolds in the Presence of a Galactopyranose Matrix by Sol-Gel Process

**DOI:** 10.3390/polym15030478

**Published:** 2023-01-17

**Authors:** Humberto Alejandro Monreal Romero, Teresa Pérez Piñon, Diana Sagarnaga, Raquel Duarte Rico, Alfredo Nevárez Rascón, Carlos Alberto Martínez Pérez, Dagoberto Pérez Piñon, Juan Pablo Flores de los Ríos, Mario Sánchez Carrillo, José Guadalupe Chacón-Nava

**Affiliations:** 1 Department of Biomaterials Science and Nanotechnology, University of Chihuahua (UACH), Avenue University, Chihuahua 31000, Mexico; 2 Institute of Engineering and Technology U.A.C.J., Avenue of Charro # 610 Cd. Juárez, Chihuahua 32310, Mexico; 3 Department of Polymers and Biomaterials, University of Chihuahua (UACH), University Circuit Campus II, Chihuahua 31110, Mexico; 4 Department Metal-Mechanical, National Technological of Mexico-Technological Institute of Chihuahua, Technological Avenue 2909, Chihuahua 31130, Mexico; 5 Advanced Materials Research Center, S.C. (CIMAV) and National Nanotechnology Laboratory, Avenue M. Cervantes 120, Industrial Complex Chihuahua, Chihuahua 31136, Mexico

**Keywords:** titanium dioxide, scaffolds, sol-gel process, galactopyranose

## Abstract

In this work, titanium dioxide scaffolds were synthesized. Titanium isopropoxide (IV) was used as a precursor in its formation, using a polymeric network of galactopyranose as a template. The powder sample obtained was evaluated by scanning tunneling microscopy (STM), transmission electron microscopy (TEM), X-ray diffraction (XRD), Brunauer-Emmett-Teller (BET) analysis, and thermal gravimetric analysis (TGA-DTA). According to the results, it was found that these scaffolds can be successfully synthesized in solution using the sol-gel method. The synthesized scaffolds have diameters from 50 nm with porosity of approximately 0.3–10 nm. Important parameters, such as pH and the concentration of the metallic precursors, were optimized in this solution. The values of maximum average roughness R(max) and roughness value (Ra) were 0.50 and 1.45, respectively. XRD diffraction analysis shows the formation of crystalline phases in the TiO_2_ scaffold at 700 °C. The use of biological polymers represents an alternative for the synthesis of new materials at low cost, manipulating the conditions in the production processes and making the proposed system more efficient.

## 1. Introduction

Several materials have been used in the past with a progressive increase in the number of industrial applications, such as nanoparticles for photocatalysis, hydroxylation processes, nanostructures as scaffolds, nanospheres, and nanoflakes, among others [1,2,3,4]. One of the most interesting nanofabrication processes is related with the exposition of TiO_2_ between several metallic precursors, such as manganese oxide, aluminum, SiO_2_, and niobium for the synthesis of coatings [5,6,7,8]. TiO_2_ scaffolds have been studied for the synthesis of biomaterials using the freeze-drying method [9]. Another example regarding the formation of nanostructures is the use of TiO_2_ scaffolds by the application of titanium hydroxide and salt matrix [10]. Among other things, these structures can be synthesized using linear polysaccharide and poly(vinyl alcohol) [11,12]. Because of the need to form new compounds at the nanoscale level, it has been made possible to explore new methods, such as nanoemulsions, sol-gel technique, and mechanical activation [13,14,15]. Molecular self-assembly processes have been used as a strategy to design nanostructures in combination with various organic molecules, such as DNA, chitosan, and extracellular matrix, among others [16,17,18,19]. A wide variety of techniques have been developed especially for the generation of materials with special characteristics, such as porosity, bioavailability, and biocompatibility [20,21]. In this way, the focus on the choice of substrate is based on its structural components to determine the degree of degradation, mechanical strength, and biological activity [22,23,24,25,26]. The generation of scaffolds provides insight into the mechanical strength characteristics of tissues, which lead to highly efficient functioning based on a system as a platform for cells growth [27]. In this way, various strategies have been created to stimulate the antibacterial properties of TiO_2_ scaffolds in the presence of PLGA [28]. Among other things, the osteogenic ability of Ti has been studied to assess the effects of osteoblastic activity on this material [29]. Likewise, the analysis of the surface of Ti compounds to improve the contact of biomaterials has been evaluated by means of techniques, such as plasma and anodic oxidation [30,31,32,33,34,35,36,37,38]. Some studies have found an inhibition effect on cancer cell proliferation using Ti scaffolds and nanoclusters, as well as chemiresistive sensors for cancer treatment, stem cell differentiation, liposome structural stability, among others [39,40,41,42,43,44,45]. The application of Ti nanostructures has been studied to generate biomaterials and understand their behavior related to osseointegration phenomena, effects of corrosion in body fluids, mechanical strength in orthopedic devices, etc. [46]. Another example of the application of scaffolds is related to their high antimicrobial capacity, because the presence of pathogenic microorganisms, can cause implant failure or proliferation of infections [47]. In tissue engineering, TiO_2_ scaffolds are widely used for bone regeneration, in combination with polymers such as chitosan, which also exhibits antimicrobial and biocompatibility properties and are prepared by different methods, such as freeze-drying, lyophilization techniques, and coatings, among others [9,48,49].

In the present work, we have synthesized TiO_2_ scaffolds using the sol-gel method. The resulting scaffolds disclosed a small size and homogeneous orientation. The aim of this study was to determine the capacity of the scaffolds to integrate into pork bone.

## 2. Materials and Methods

### 2.1. Synthesis and Characterization of TiO_2_ Scaffolds

The experimental procedure for the synthesis of TiO_2_ scaffolds through the sol-gel method was carried out as follows. In the first step, the compounds were prepared with the addition of 50 mg of Agarose (1–3 linked 𝛽-Dgalactopyranose and 1,4 linked 3,6 anhydro-𝛼-Lgalactopyranose (99% Sigma-Aldrich, St. Louis, MO, USA) powder into an ethanol aqueous solution made of 200 mL of ethanol to 99.8% (Sigma-Aldrich) and 50 mL of distilled water. Afterwards, this aqueous solution containing the galactopyranose polymer was heated at 35 °C for about 2 min in a Corning PC-351 hot plate (One Riverfront Plaza, Corning, NY, USA), and then, it was left at room temperature for 24 h at pH 11 under a magnetic stirrer. Afterwards, once the sol is formed, 25 mL of titanium isopropoxide Ti [OCH (CH_3_)_2_]_4_ −97% (Sigma-Aldrich) 1 M was added and was dried at room temperature for 2 h for the gel formation. In this way, the metallic precursors interact, forming a network of electrostatic unions for the formation of scaffolds. In a third step, the gel was washed with bidistilled water several times to eliminate any amount of ethanol. After this, the gel was placed in a laboratory electrical kiln at 700 °C for 1 h. To characterize the morphology of compounds, a scanning tunneling microscope Nanosurf Easyscan 2 instruments (Liestal, Switzerland) equipped with Pt/Ir tips (BT00400) was used, and the images were processed using an Easyscan 2 image software version 1-6-0-0. Additionally, a characterization by transmission electron microscopy was carried out in a Phillips CM-200 TEM (Amsterdam, the Netherlands) with an acceleration voltage of 200 kV. The TGA-DTA analysis of the scaffolds was carried out in a DTA-TGA TA instrument (New Castle, DE, USA) using a heating rate of 10 °C/min in air, and the powder crystalline phase was identified by X-ray diffraction (XRD) in a Phillips X’PERT X-Ray diffractometer using a CuK (α) source at 0.1542 nm. The determination of surface area was made by using the BET method. Fractal dimension, hole profile and R2 correlation studies were performed using Mountains Lab USA software version 9 (Digital Surf, Besançon, France). transilluminator (Fisher Scientific, Madrid, Spain) in agarose gel electrophoresis.

### 2.2. Preparation of TiO_2_ Scaffolds by Agarose Gel Electrophoresis

A 0.88% (*w*/*v*) solution of agarose gel was prepared in a TBE buffer (tris, borate, EDTA buffer 1X). For this purpose, 266 mg of agarose were taken and 30 mL of TBE 1X buffer were added, heating in a microwave oven until the agarose was dissolved then proceeded to polymerization, 3 µL of an ethidium bromide solution (10 mg/mL) (Sigma-Aldrich) was added. In order to avoid the formation of bubbles, this solution was gently mixed, and subsequently deposited on the gel until full polymerization. Afterwards, 20 mg of the polysaccharide in the absence of TiO_2_ were added in lanes 1, 3, and 4, whereas in lane 2 the powders containing the TiO_2_ scaffolds were placed. Later, a 280 mV voltage was applied for 1 h in the horizontal electrophoresis system and the gel was visualized under ultraviolet light (230 nm wavelength).

### 2.3. Biomineralization Test of TiO_2_ Scaffolds in SBF

For the biomineralization test, a simulated body fluid (SBF) solution was prepared by mixing the following laboratory reagents at the indicated ion concentration (mM) in ion-exchanged and distilled water: Na^+^ 142.0, K ^+^ 5.0, Mg ^2+^ 1.5, Ca ^2+^ 2.5, Cl^−^ 147.8, HCO_3_^−^ 4.2, HPO_4_ ^2−^ 1.0, and SO_4_ ^2−^ 0.5. For the TiO_2_ scaffolds tests in SBF, 1 gr of the powders containing the scaffolds and 200 mg of pork bone previously treated were added to 1 mL of acetone (99.9%, Sigma-Aldrich). This mixture was centrifuged at 5000 rpm for 15 min in an Eppendorf 5424 microcentrifuge. Later, the powders were compacted at 4500 psi forming pellets of 0.5 cm in diameter and 5 mm thickness. Once the pellets formed, they were immersed in the SBF solution at 36.5 °C for 4 weeks. After this time, the pellets were washed several times with distilled water and dried in an atmosphere of 95% air and 5% CO_2_ for 24 h, and then analyzed using a scanning tunneling microscope Easyscan Nanosurf 2, EDX spectroscopy, and power spectrum density (PSD) analysis using a Mountains Lab Premium 8.0 surface analysis software (Digital Surf).

### 2.4. UV–Vis Absorbance

A solution containing 0.5 g of TiO_2_ scaffolds in 50 mL tubes with deionized water was prepared. After the samples were placed in quartz cuvettes, absorbance measurements were carried out using a UV–Vis spectrophotometer ranging from 200 to 800 nm.

## 3. Results and Discussion 

### 3.1. Characterization of Scaffolds Using Scanning Tunneling Microscopy (STM)

**Figure 1:**
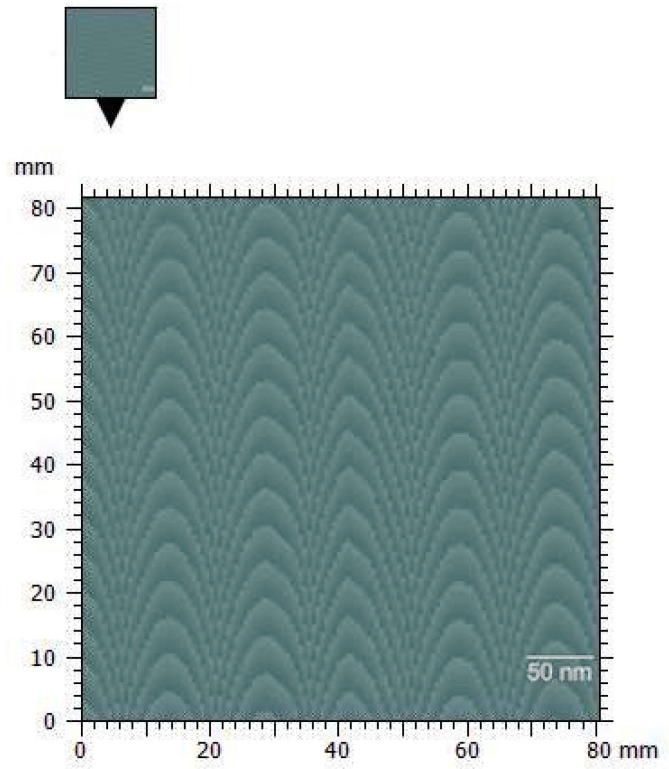
STM image of TiO_2_ scaffolds.

**Figure 2:**
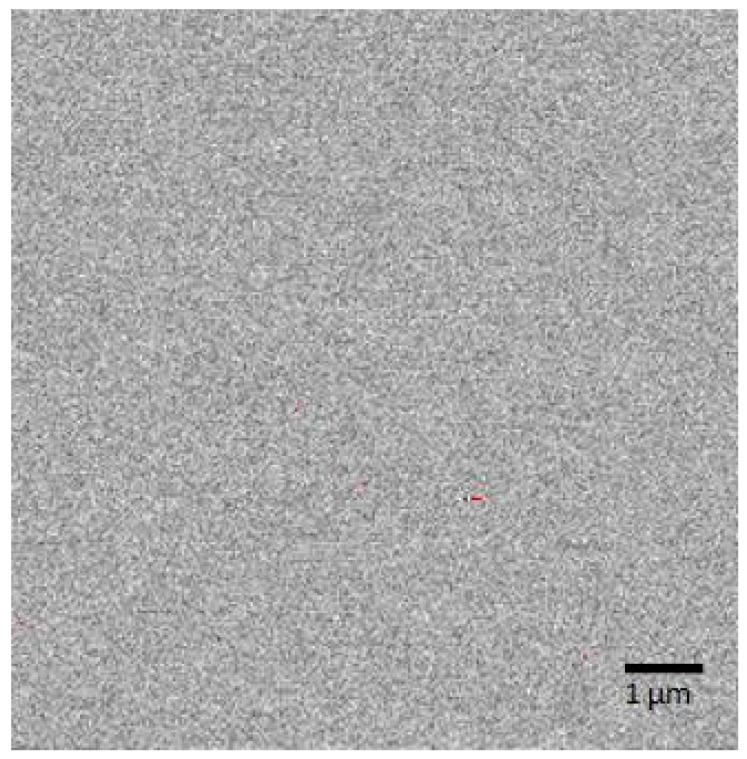
Microstructural analysis of TiO_2_ scaffolds.

The morphology of the scaffolds was investigated by scanning tunneling microscopy (STM). Figure 1 shows the morphology of TiO_2_ scaffolds of 50 nm in diameter with a porosity of about 10 nm, with the inset image being representative at the atomic level. Scaffolds produced by the sol-gel method showed the classical microstructure of the conventional thermally synthesized scaffolds and the presence of small particles, which can be observed in Figure 2. The surface of the TiO_2_ scaffolds is rather homogeneous. These images suggest that the electrical behavior of the tunneling junction composed of TiO_2_ scaffolds is dominated by an electron-transfer mechanism, characteristic of isolated scaffolds. In another work, the electrical behavior of scaffolds in bone tissue was analyzed [50]. The results obtained are compatible with this study.

### 3.2. Characterization of Scaffolds by Transmission Electron Microscope (TEM)

**Figure 3:**
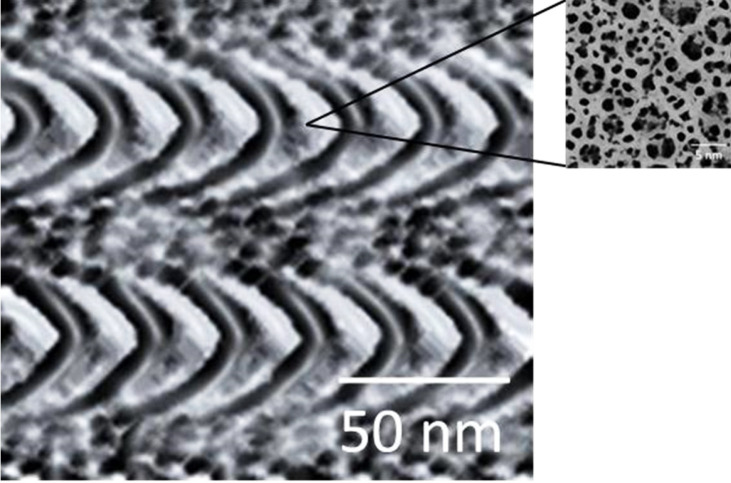
TEM image of TiO_2_ scaffolds.

**Figure 4:**
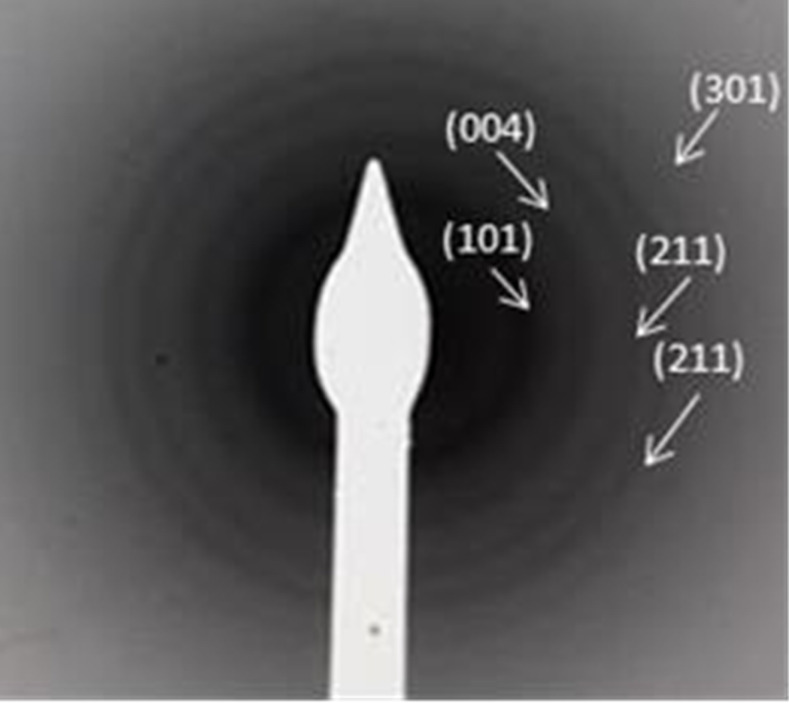
SAED pattern of TiO_2_ scaffolds.

Figure 3 shows the scaffolds characterized by transmission electron microscopy (TEM), a greater definition of the nanostructures can be observed which are composed of two compartments, a compartment with a smooth surface with a thickness of 3 nm and another compartment with a thickness of approximately 10 nm. The ranges of pore sizes in the second compartment are between 0.3 and 5 nm. The development of porous materials for applications in biomedicine is very important, since the pore walls have a large area that allows the incorporation of various nutrients. Another application is a greater stimulus for the formation of osteoblastic cells, causing an increase in the mechanical strength of the pore walls. In other research, the pore size between 100 and 700 µm, has been reported [50]. We suggest that the pore size of scaffolds can have multiple applications, such as functionalization of intercellular adhesion proteins or cellular adhesion, inhibiting the proliferation of tumor cells that can reach organs or systems. Other areas of application would be at the industrial scale for the development of devices that inhibit environmental pollution of wastewater, or in catalytic processes and biomass conversion. Figure 4 shows the selected area electron diffraction (SAED) patterns in which the hkl planes, (101), (004), and (211) correspond to the anatase phase, the planes (211) and (301) corresponding to the rutile phase are also observed. The intensity of the rings shows the complete crystallinity of the TiO_2_ scaffolds and the formation of the phases, which was confirmed with the X-ray diffraction analysis. Among other things, the TEM analysis was used to learn the texture details of the scaffolds additional to the STM studies.

### 3.3. Roughness Profile of the TiO_2_ Scaffolds

**Figure 5:**
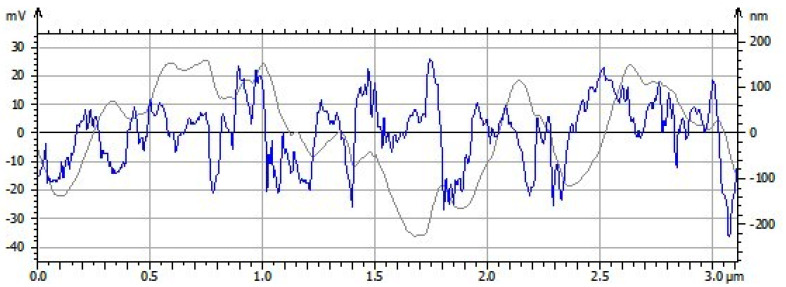
Roughness profile of TiO_2_ scaffolds.

Figure 5 shows the roughness profile of the TiO_2_ scaffolds with a sample length of 3.114 µm. The results show a maximum roughness (Rmax) average value of 0.50 nm, which is characteristic of the difference between the maximum peak height and the depth of the valley, with an average roughness (Ra) value of 1.45 nm. In this graph, it can be seen that the undulation accompanies the general shape of the profile (blue line) and some inclination is perceived that is interrupted for each sample compartment (gray line). In this way, the measurement modules were characterized based on the depth of roughness and maximum roughness as correct parameters in the roughness profilometry of the TiO_2_ scaffolds. The roughness characteristics are determined by various factors, such as heat treatment, the contraction of the polysaccharide fibers by the evaporation process, as well as the chemical interaction of the elements during the synthesis. In this context, it is important to highlight that it is complicated to control the level of roughness in order to have a really perfect surface, since the mechanism of molecular self-assembly is driven by processes of supramolecular chemistry and electrostatic interactions.

### 3.4. X-ray Diffraction 

**Figure 6:**
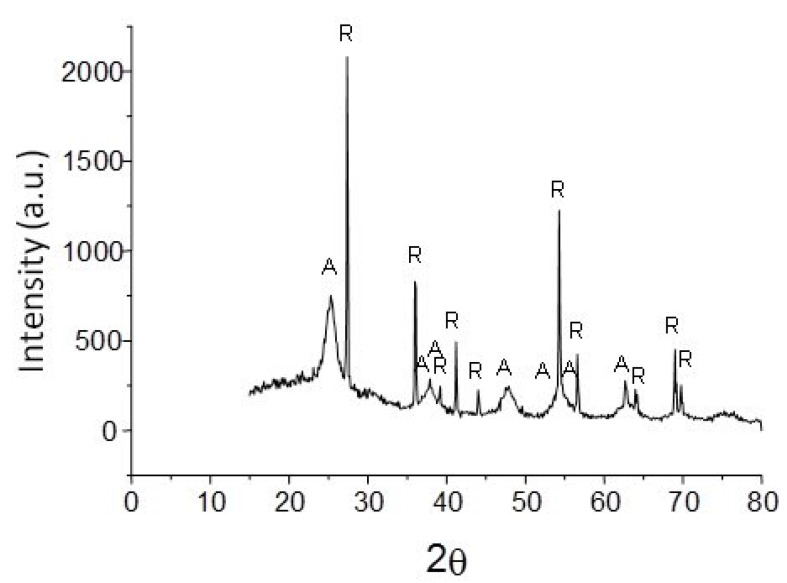
X-ray diffraction pattern obtained from the TiO_2_ scaffolds, A—anatase, R—rutile.

Figure 6 shows the diffraction pattern of the powder obtained at room temperature. Here, peaks at 25°, 37°, 38°, 48°, 54°, 55°, and 63° 2ϴ that correspond to the anatase phase can be seen. Additionally, peaks at 27.5°, 36°, 39°, 41°, 44°, 54°, 56.5°, 64°, 69°, and 70° 2ϴ corresponding to the rutile phase can be seen. In this case, the XRD pattern shows that the powder sample was crystalline. The diffractogram shows the crystallinity of the sample and the intensity of the reflections, indicating a separation of the amorphous part followed by a greater crystalline part. The difference is quite noticeable with respect to the first reflection, because the sample is mostly amorphous, therefore it does not present a defined pattern. Afterwards, reflections are clearly identified, indicating the loss of amorphous material in the sample. In this way, the degree of crystallinity obtained, is in a range of 87 to 90% of rutile and 66% of anatase approximately. The degree of crystallinity was made by measuring the intensity of the reflections in the diffractogram. In the proposed system, the rutile phase presents a thermodynamic stability, however, the presence of anatase and the polysaccharide do not inhibit the crystallization of TiO_2_ in the presence of temperature, likewise the effect of temperature does not allow the transformation of the anatase phase to rutile, in this way we believe that the polysaccharide plays an important role in the maintenance of each crystalline phase due to the self-assembly process. For this reason, the utility for forming structures with the anatase and rutile phases lies mainly in the fact that the presence of rutile can reduce the production of free radicals formed by the anatase phase during the sintering processes, thereby avoiding the degradation of organic molecules.

### 3.5. TGA and DTA Analysis 

**Figure 7:**
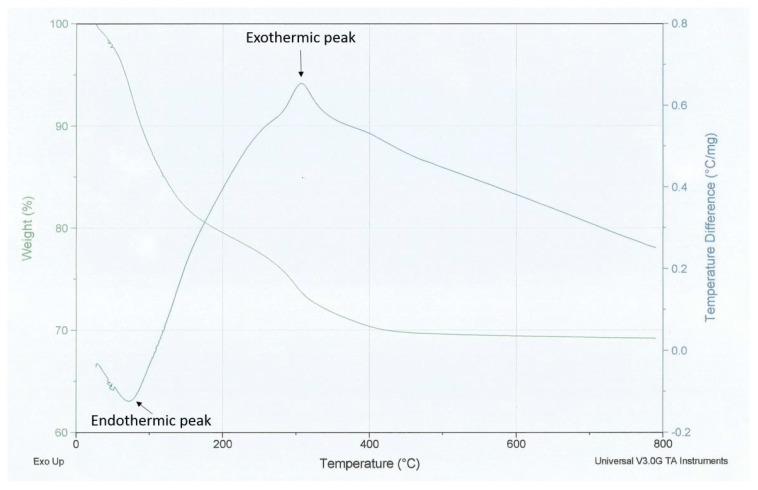
TGA-DTA analysis of the TiO_2_ scaffolds.

The TGA curve shows a water weight loss in the temperature range from 80° to 180 °C, see Figure 7. At about 40 °C, water weight losses do not exist due to the oxidation of residual organic groups from the surface of the polymeric matrix. From 180 °C up to about 600 °C the precursor is degraded. Above 600 °C, the TiO_2_ phases (anatase and rutile) remain. The DTA curve shows an endodermic peak at about 100 °C, and exothermic peaks at about 280 and 450 °C. These results show that the anatase to the rutile phase transition of TiO_2_ is not due to the surface reduction in anatase, but rather to the addition of the polysaccharide that can inhibit the phase transitions, which does not cause the formation of oxygen vacancies at the temperature of formation of the scaffolds. 

### 3.6. Brunauer-Emmett-Teller (BET) Analysis

The surface area of the sample was determined using the BET method. The scaffold pore size was calculated from the surface area following the equation DBET = 6000/(ρS), where DBET is the equivalent particle size in nanometers, ρ is the density of the particle in g/cm^3^, and S is the specific surface area in m^2^/g. From BET analysis, a specific area of 230 m^2^/g was obtained. Thus, the average equivalent scaffold size is 50 nm. In the literature, a surface area of 13,627 m^2^/g has been reported [51].

**Figure 8:**
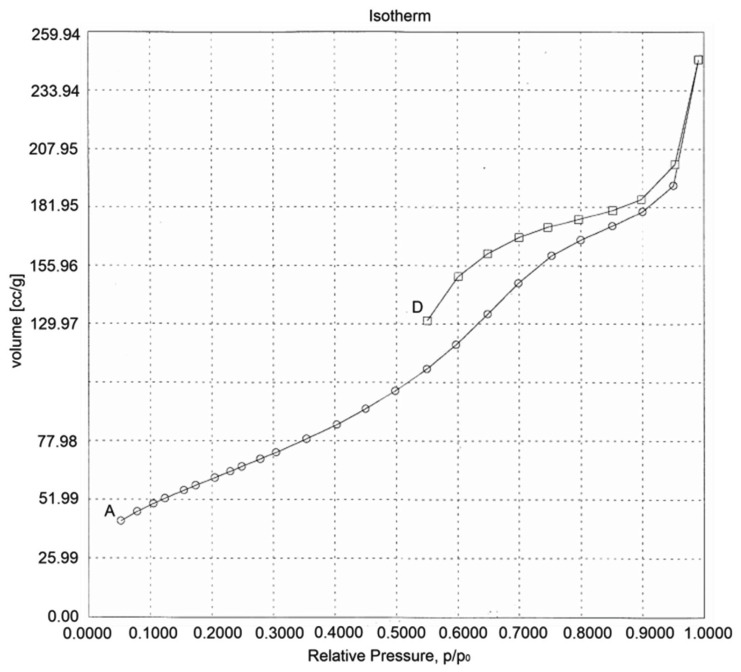
Brunauer-Emmett-Teller (BET) analysis of TiO_2_ scaffolds, A—adsorption, D—desorption.

Figure 8 shows the isotherm of TiO_2_ scaffolds disclosing minor differences among the adsorption and desorption processes with pore sizes of 0.3–10 nm, reflecting a homogeneous characteristic on the surface of the first compartment, helping to establish that the type of graph is type 2. This type of graph explains the adsorption mechanism, which is in multilayers, this makes that the condensation of the polysaccharide take place in the pores of the scaffolds, is in this place, where the interactions of forces of van der Waals can be carried out by active sites, in which the polysaccharide retains its identity.

**Figure 9:**
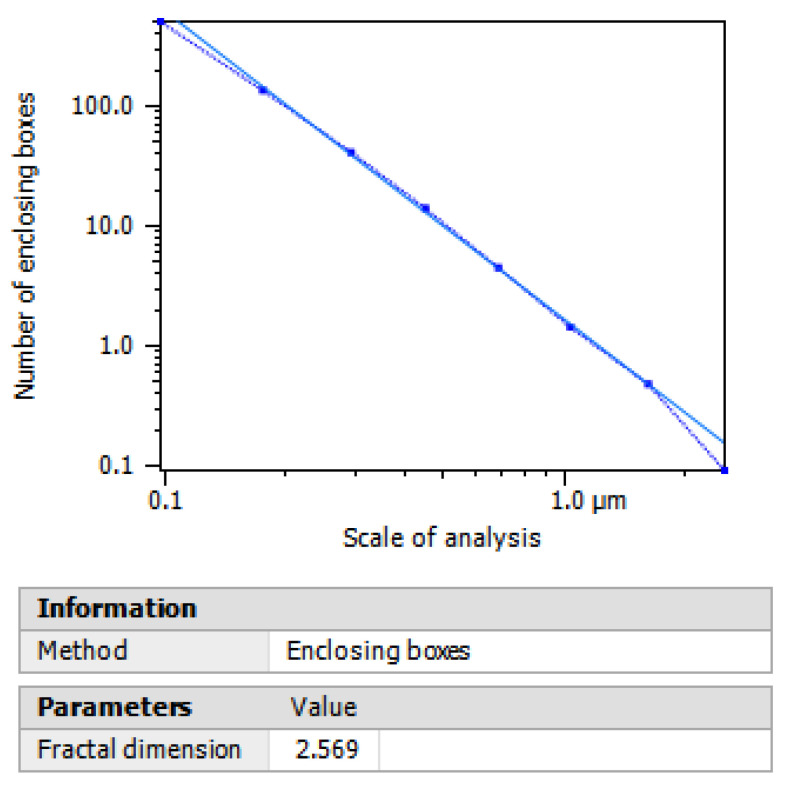
Fractal dimension of TiO_2_ scaffolds.

Figure 9 shows the fractal dimension analysis. The scaffolds were analyzed using the equation: log S = D log L → D = log S / log L. Where S is the fractal size, L is the measurement scale (2.246), and D is the unknown fractal dimension (8). The scaffolds did not present any irregularity in their morphology, which indicates a positive correlation in the values of the fractal dimension, whose value is 2.569, meaning that there are no degrees of deformation, which confirms the degree of symmetry established by the Euclidean geometry for regular shapes showing a linear behavior in its length. These results are similar to those reported by Fostad et al., who establish a fractal dimension of 2.35 [52].

**Figure 10:**
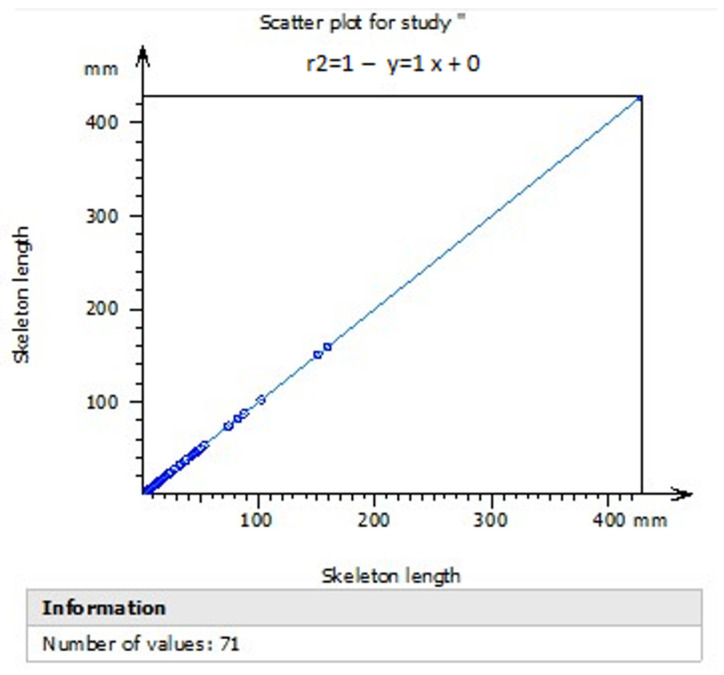
Determination coefficient R2.

Figure 10 shows the correlation coefficient R2 with a value of 1, which is consistent for the proposed model to be replicated. Likewise, the calculation of the mean presented a value of 31.07 and a standard deviation of 59.95 of the boundary perimeters of the fractal dimension of the scaffolds. These results show a high regularity in the perimeters that avoid variability and errors when measuring fractal characteristics such as repeated patterns, self-similarity, and dimension, see Table 1.

**Figure 11:**
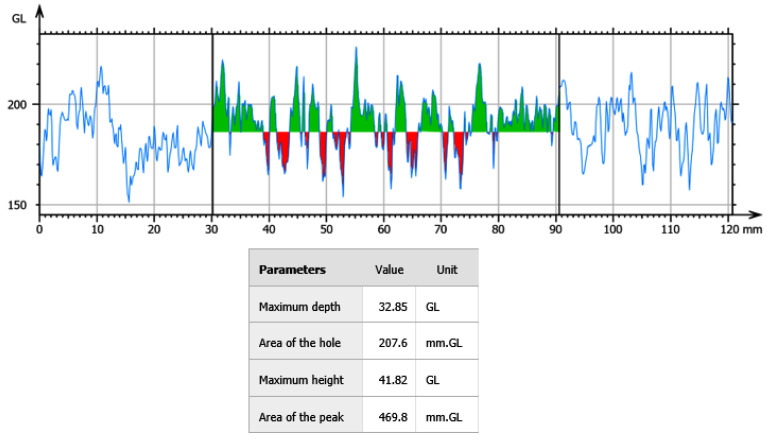
Hole profiles of TiO_2_ scaffolds.

Figure 11 shows the profile of the hole formation of the scaffolds. The section below the middle line (red) corresponds to the holes, and the section above the middle line (green) corresponds to the first layer of TiO_2_ which is pushed outside at the time of hole formation, having, as its base, the polysaccharide matrix with a maximum depth of 32.85 gl and a hole area of 207.6 mm × gl. In this way, the polysaccharide allows homogeneous separation during the electrostatic interaction mechanism between the H and O_2_ residues of TiO_2_ and the polymeric matrix. During the sintering processes, the degradation of the organic material promotes the formation of pores within the compound, this allows tissue uptake to increase through cell proliferation [53].

### 3.7. Characterization of TiO_2_ Scaffolds in Agarose Gel Electrophoresis

**Figure 12:**
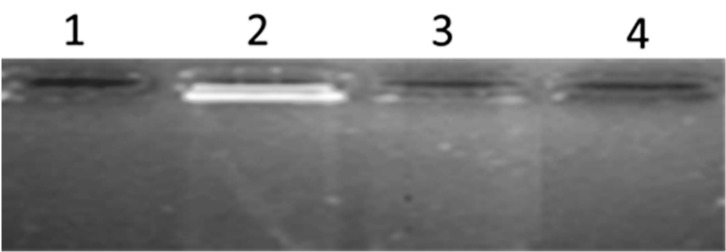
Agarose gel electrophoresis of TiO_2_ scaffolds.

Figure 12 shows an agarose gel electrophoresis of powders containing the TiO_2_ scaffolds. In lane 1, 3, and 4, polysaccharide powders were deposited in the absence of TiO_2_, whereas in lane 2 the TiO_2_ scaffolds in the presence of the polysaccharide were deposited. Here, the fluorescence of the TiO_2_ scaffolds complex can be clearly seen showing a short superior band and a long lower band. The shape of the bands are due to the horizontal structure of the gel cavity, where the powders are deposited. It is important to comment on the effect of applying a voltage of 280 mV during the electrophoresis run, because the bands manage to leave the rail towards the positive pole of the electrophoresis chamber, which shows that the TiO_2_ scaffolds complex has a negative charge. The fluorescence is given by the cationic intercalation of the ethidium bromide and the anionic charges of TiO_2_ scaffolds complex in the presence of the polysaccharide. In this way, the cationic intercalating agent is electrostatically attracted to the polyanionic system. The ligand forms a weak electrostatic bond with the oxygen of the TiO_2_, and, in this way, the aqueous solution acts as a hydrophobic medium in such a way that the ligand can move through the scaffolds causing a fluorescence effect with an ultraviolet light absorption of 230 nm. Other studies using agarose gel electrophoresis with Ti have been explored [54,55].

### 3.8. Characterization of TiO_2_ Scaffolds in SBF by STM 

**Figure 13:**
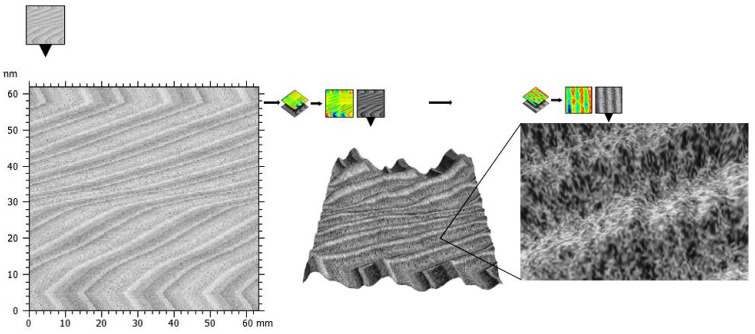
TiO_2_ scaffolds in presence of pork bone.

STM analysis revealed that the TiO_2_ scaffolds have been oseointegrated. According to the results obtained, pore sizes in the range of 1–50 nm have been reported in the literature [56,57,58]. Figure 13 shows the scaffolds in the bone matrix, which disclosed a homogeneous morphology. It is important to note that the surfaces of the scaffolds were covered by bone tissue. These results demonstrate the benefits of developing a non-invasive technique through which it is possible to obtain the restoration of the functional activity of the damaged bone tissue. Thus, TiO_2_ scaffolds are important to define the general behavior when the skeletal system interacts with various nanostructures. The strength of bone depends on anisotropic values of ultimate strength (tension, compression, shear), elastic modulus, and Poisson’s ratio, as well as the stress-strain behavior. Therefore, the synthetized scaffolds might play an important role in mechanisms, such as bone resorption. This suggests the possibility that the TiO_2_ scaffolds would have high affinity to calcium substrates and, therefore, would be more effectively retained in the bone.

From Figure 13, it appears that the STM on biological samples can achieve the kind of lateral resolution only on ordered sets of scaffolds, where the destructive effects of lateral forces are less on individual biological molecules. From the present experimental results, it can be said that the study of scaffold–bone interactions depends on three factors: (a) the yield of the reaction, that is, the number of scaffolds that interact with the bone substrate; (b) sample purity: purification of the two essential ingredients, i.e., scaffolds and bone, is very important. Pure samples should be used because, otherwise, we would have a lot of specific interactions that could mask the real situation; and (c) the adsorption performance: this last factor is important for the interaction of molecules into liquid. In adjusting the above parameters, it is very important to address STM interaction issues. However, despite the problems that may arise to have a proper adjustment, STM is presented as an alternative technique when mapping material interactions and possibly bone–scaffolds interactions. Figure 13 shows the scaffolds as part of bone tissue, thereby demonstrating compatibility and biological activity prevalent between bone and the material. The present study also suggests that the increase in bone functionality, might be due to the similarity of the structure of scaffolds and bone, and, in this manner, the surface energy of the nanostructures promotes quick adhesion. Additionally, similar studies have related an increase in TiO_2_ bioactivity in rat bones [59]. The insertion of atoms of different species interspersed introduces additional states around the Fermi level, meaning that scaffolds increase their electron emission. The results show that the insertion of nanostructures in bone can withstand the forces caused by STM derived from electrostatic charges that cause difficulties in topographic studies of bone. We believe that this type of insertion (nanostructured-bone) occurs because the oxygen present in the scaffolds reacts with the calcium group in the bone. The interest in the realization of the scaffolds, is the formation of porous structures that improve the absorption and distribution characteristics of different organic molecules, such as blood, tissue, and various nutrients, among others.

### 3.9. EDX Analysis 

**Figure 14:**
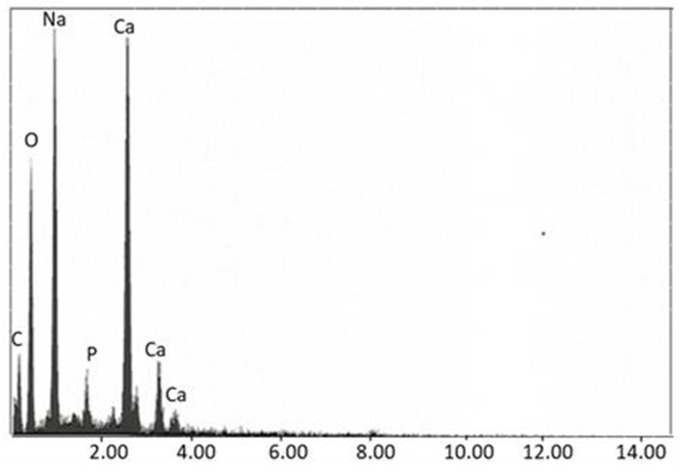
EDX spectrum of TiO_2_ scaffolds with pork bone in SBF.

Figure 14 shows the EDX spectrum of the scaffolds with bone tissue, indicating the presence of Ca, P, Na, and O in the sample.

### 3.10. Power Spectrum Density (PSD)

**Figure 15:**
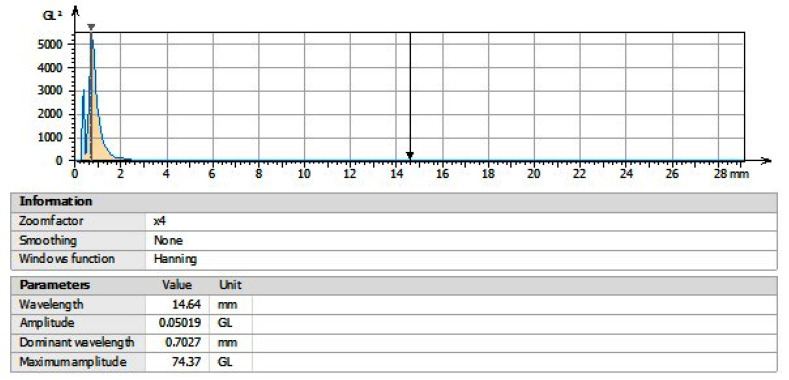
Average power spectrum density of scaffolds in presence of pork bone.

Figure 15 shows the power spectral density (PSD) to evaluate the spectral energy distribution. Here, a series of displacement values (in millimeters) can be noted. The dominant wavelength is represented with a maximum peak of 0.7027 mm in relation with a maximum amplitude of 74.37 GL, and a curve drop is observed at approximately 2.8 mm, with normalization at 14.64 mm. These results represent the spectral behavior, with the x-axis given in wavelengths and the z-axis given in amplitudes, and demonstrate that the signals have different frequency values, which are related to the different degrees of freedom of the system. Further, this indicates that the mechanism of scaffolds formation is one of stochastic nature. The measurement of the spectral distribution has been evaluated in other study with values of 15 nm as the maximum peak and an amplitude of 0.5 µm [60].

### 3.11. UV–Vis Absorbance

**Figure 16:**
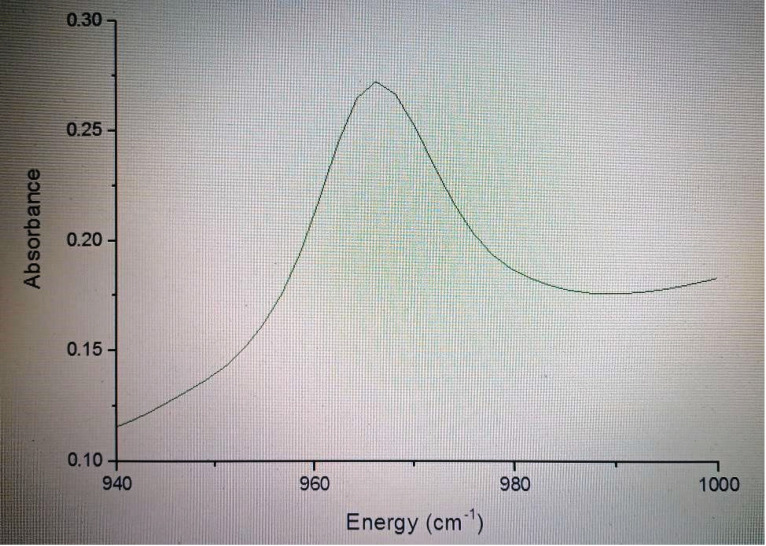
UV–Vis spectrum of TiO_2_ scaffolds.

Figure 16 shows the UV–Vis absorption spectrum. The spectral data show a cut-off value at about 980 cm^−1^ and, in particular, the absorbance value is minor. For this reason, the role of the polysaccharide network can be an important factor in the energy increase, which can be stabilized by the hydrogen bonds present in the polysaccharide matrix with the TiO_2_ structure. In addition, it is very difficult to establish the visible light absorption of the scaffolds because of the Ti in solution, which could be found in low amounts due to the electrostatic layer-by-layer self-assembly method. From the diffuse reflectance spectrum, it is not possible to evaluate this aspect associated with the system under study, because in dispersive media the optical path traveled by the light is difficult to establish, since the decrease in radiation is due to absorption and dispersion. In this way, the absorption behavior coincides with a work reported by Kangqiang et al. made with Ti and other materials [61]. The measured band gap of the scaffolds was 2.87 eV. These data are important because, in this way, the scaffolds may play an important role in the absorption of other types of light (such as visible light), reducing the inconvenience of having a large TiO_2_ band gap when it is not linked with other materials. In this way, the scaffolds would be better conductors for the visualization of structures, such as bones, teeth, lungs, and liver, among others, through X-ray application, due to its small band gap, i.e., a low band gap implies higher intrinsic conduction. The band gap energy was calculated as follows: Band Gap Energy (E) = hC/λ., where h is the Plank constant (6.626 × 10^−34^ J s), C is the speed of light (3.0 × 10^8^ m/s) and λ is the minimum absorption wavelength.

## 4. Conclusions 

We have reported the formation of TiO_2_ scaffolds, in a range of 50 nm in diameter obtained at low cost and as an easy route for the fabrication of nanostructured materials. Among others, these materials can be used in areas, such as materials science, bioengineering, nanomaterials for medicine, electronic systems, etc. The analysis of the measurement of the scaffolds perimeters and the correlation parameter R2, show the presence of fractal characteristics, which indicates that the fractal dimension is not affected by geometric symmetry. The XRD results confirmed that the compounds obtained show crystalline phases of the metallic precursors at 700 °C. In summary, TiO_2_ scaffolds were examined to assess their adhesion viability in polysaccharides and pork bone. Furthermore, the materials’ characteristics are important so that the compounds can be functionalized on the surface of TiO_2_. In this way, it would be possible to analyze the effect of several factors, such as pH, temperature, applied voltage, and molecular self-assembly. In addition, the UV–Vis analysis indicated that the scaffolds present a remarkable reduction in the band gap disclosing a value of 2.87 eV. The results confirm that the formation of TiO_2_ scaffolds, is appropriate to be used as platforms for the integration of cells and tissues, due to the physical and chemical characteristics presented. Finally, in other studies of these materials, we are currently investigating aspects related to their mechanical properties and corrosion behavior.

## Figures and Tables

**Table 1:** Mean and standard deviation of TiO_2_ scaffolds.

Statistical Summary					
Parameters	Unit	Mean	Std Dev	Min	Max
Projected Area	mm^2^	31.07	59.95	0.03644	432.2
Skeleton Length	mm	29.56	57.89	0.5080	428.5

## Data Availability

Due to an institutional policy, the data is not available, however it can be requested through the address of the corresponding author.
